# From Neurophobia to Neuroanxiety: Between Metaphor, Semantics, and Pedagogy

**DOI:** 10.1212/NE9.0000000000200271

**Published:** 2025-10-29

**Authors:** Renato Faria da Gama, Crisóstomo Lima do Nascimento, Camila Castelo Branco Pupe

**Affiliations:** 1 Centro Multidisciplinar UFRJ Macaé, Niterói, Rio de Janeiro, Brazil;; 2 Programa de Pós-Graduação em Cognição e Linguagem da UENF, Niterói, Rio de Janeiro, Brazil; and; 3 Programa de Pós-Graduação em Neurologia e Neurociências da UFF, Niterói, Rio de Janeiro, Brazil.

The recent Viewpoint by Tan, Lin, and Tan (2025) revisits the enduring concept of *neurophobia*, proposing *neuroanxiety* as a more accurate descriptor of medical students' responses to neurology. Before adopting a new label, it is worth retracing the history of the concept, its semantic load, and the consequences of renaming a construct that has guided research and reform for 3 decades.

## Historical Emergence and Consolidation

Concerns about the cognitive weight of neurology predate formal terminology. In his 2013 Presidential Plenary at the American Academy of Neurology, Jerome Posner emphasized that neurology, unlike most specialties, rests heavily on listening and reasoning—an implicit acknowledgment of why students often perceive it as daunting.^1^ This apprehension was earlier crystallized by Jozefowicz,^2^ who coined *neurophobia* as “fear of the neural sciences and clinical neurology due to students' inability to apply basic science to clinical situations.”

Over subsequent decades, studies in North America, Europe, Asia, Africa, and Latin America confirmed the phenomenon and cemented *neurophobia* as a transnational category of medical education.^1^ A 2017 editorial in *Arch Med Health Sci* expanded the semantic scope of neurophobia to include anxiety, aversion, and disinterest, anticipating a multidimensional affective profile.^3^ In 2022, Andrews and Riggs^4^ argued that the word *phobia* is misleading under DSM-5 criteria and suggested *neuroavoidance*. Tan et al.^5^ (2025) now propose *neuroanxiety*, supported by a systematic review and a survey showing that anxiety was more prevalent than fear, with avoidance as a frequent correlate.

## Semantics and Nosology

Words are not inert descriptors; they presuppose meanings and guide inferences. In psychiatric nosology (DSM-5), *specific phobia* denotes irrational, disproportionate fear with persistent avoidance. *Anxiety* refers to anticipatory apprehension, potentially adaptive, while *avoidance* designates behavioral strategies rather than underlying affect. Semantically, then, *phobia* pathologizes, *anxiety* contextualizes, and *avoidance* operationalizes behavior. Each term thus frames differently how educators and students conceptualize the challenge.

## The Case for and Against Renaming

### For Change

*Neuroanxiety* resonates more closely with students' lived experiences of anticipatory apprehension and aligns with psychiatric distinctions between fear and anxiety. It avoids the pathologizing overtones of *phobia* and directs attention to interventions aimed at self-efficacy, stress regulation, and curricular design.

### Against Change

*Neurophobia* has become a powerful heuristic—mobilizing awareness, enabling standardized measurement, and sustaining international comparability. Abandoning it risks fragmenting a mature literature and undermining longitudinal benchmarking. As Shelley^3^ illustrates, the term has already evolved metaphorically to encompass anxiety and aversion; reinterpretation may achieve clarity without rupture.

## A Dimensional Synthesis

An integrative model may be more fruitful than substitution. Retain *neurophobia* as the umbrella construct, preserving continuity. Within this framework, *neuroanxiety* can denote the predominant affective dimension most responsive to intervention while *avoidance* can be recognized as its behavioral manifestation. This layered framework resonates with the integrative view advanced by Gama,^1^ who argued that neurophobia is multifactorial, shaped by symbolic-affective dissonance, pedagogical modality, and cognitive overload.

## Conclusion

The debate over neurophobia is more than lexical. It exemplifies how language not only reflects but also shapes how we perceive and address educational challenges. Semantics are not trivial: the choice between phobia, anxiety, and avoidance directs both research trajectories and pedagogical strategies. Moreover, some students may not experience fear or anxiety at all, but rather a lack of intrinsic interest—a form of “neuroaversion”—that calls for different educational approaches. Whether fear, anxiety, avoidance, or disinterest, the true task is to transform apprehension into engagement. Terminology should serve that pedagogical goal—not eclipse it.
